# Preoperative serum 8-hydroxydeoxyguanosine is associated with chemoresistance and is a powerful prognostic factor in endometrioid-type epithelial ovarian cancer

**DOI:** 10.1186/s12885-015-1504-6

**Published:** 2015-07-02

**Authors:** Marjo Pylväs-Eerola, Peeter Karihtala, Ulla Puistola

**Affiliations:** 1 Department of Obstetrics and Gynecology, Medical Research Center Oulu, University of Oulu and Oulu University Hospital, Oulu, Finland; 2 Department of Oncology and Radiotherapy, Medical Research Center Oulu, University of Oulu and Oulu University Hospital, P.O. Box 22, FIN-90029 Oulu, Finland

**Keywords:** Oxidative stress, 8-OHdG, DJ-1, Epithelial ovarian cancer, Endometrioid ovarian cancer, Platinum resistance

## Abstract

**Background:**

Oxidative stress is a widely seen phenomenon in several carcinomas. Increasing evidence also suggests that it has a significant role in the development of epithelial ovarian carcinoma (EOC). 8-Hydroxydeoxyguanosine (8-OHdG) is one of the main indicators of oxidative stress and increased expression of 8-OHdG has previously been seen in EOC. DJ-1 is an oncoprotein connected to oxidative stress regulation, but its role in ovarian cancer is not well known. We investigated redox status in different histotypes of EOC by measuring serum 8-OHdG and DJ-1 concentrations and their associations with known prognostic factors.

**Methods:**

Serum samples from newly diagnosed EOC patients were collected in 1996–2009 and stored at the Department of Obstetrics and Gynecology, Oulu University Hospital. Serum 8-OHdG and DJ-1 levels were measured by using commercially available ELISA kits. Clinical data was gathered retrospectively from the patients` files. Results were analyzed by using SPSS software.

**Results:**

In total, 112 patient samples were analyzed (38 serous, 20 mucinous, 34 endometrioid and 20 clear-cell). High serum 8-OHdG levels were associated with poor overall survival (OS) (p = 0.019), poor disease-free survival (DFS) (p = 0.020), platinum resistance (p = 0.002), serous histology versus other (p = 0.033), stage III–IV versus I–II (p = 0.009) and suboptimal surgical outcome (p = 0.012). Regarding histotypes, in the endometrioid EOC group in particular, serum 8-OHdG levels were significantly associated with poor DFS (p = 0.005), suboptimal surgical outcome (p = 0.025), and platinum resistance (p = 0.007). The prognostic significance of 8-OHdG in patients with endometrioid cancer in terms of DFS was confirmed in Cox regression analysis. High DJ-1 levels were associated with high histological grade (p = 0.029) and nonsignificantly associated with serous histology vs. other histology (p = 0.089).

**Conclusions:**

An elevated serum 8-OHdG level is a significant predictor of poor prognosis, especially in cases of the endometrioid subtype of ovarian carcinoma. High 8-OHdG levels are associated with all traditional factors of poor prognosis in ovarian cancer and they also predict earlier development of platinum resistance. These results could be valuable when deciding the primary treatment mode for EOC patients.

## Background

Ovarian cancer is the seventh most common cancer worldwide and one of the main causes of cancer-related death. Usually it is diagnosed at an advanced stage when the prognosis is poor. In most cases relapse occurs within two years, the patient becomes chemoresistant and five-year survival is only 45 % at best. The known risk factors of ovarian cancer are infertility, family history, smoking and hormone replacement therapy, but the main reasons for the occurrence of ovarian cancer are still unclear. In recent years many studies have shown the role of oxidative stress in the development of ovarian and other cancers.

Evidence suggests that ovulation increases the levels of inflammatory agents, which can further lead to mutations in DNA [1–4]. Ovulation creates a void on the ovarian surface, which leads to the wound-healing process, with increased levels of inflammatory mediators and reactive oxygen species (ROS) [1–3]. It has been suggested that cancer cells from the fallopian tubes, the cervix or endometriotic lesions migrate to the ovaries and form tumors near the corpus luteum [5].

Reactive oxygen species are formed either from incomplete reduction of oxygen during cellular respiration, or following exposure to external agents such as ultraviolet light, ionizing radiation or redox-state-modifying drugs, which are factors known to be connected with carcinogenesis [6]. Because of their unpaired electrons, ROS molecules are very unstable and react easily with other molecules. They can interact directly with DNA and they can oxidize lipids and proteins, generating intermediates that react with DNA, a cascade with the potential to cause DNA mutations when antioxidant defenses are overwhelmed [6]. The hydroxyl radical (•OH) is extremely unstable and reacts rapidly with other molecules. When it attacks DNA, 8-hydroxydeoxyguanosine (8-OHdG) can be formed. Tissue expression of 8-OHdG and its serum concentrations are associated with the prognosis of several carcinomas, including ovarian cancer, breast cancer, colorectal cancer, cutaneous melanoma, non-small-cell lung cancer, diffuse large B-cell lymphoma and esophageal cancer [7–13]. In colorectal carcinomas, immunohistochemical expression levels of 8-OHdG and nitrotyrosine are significantly higher in malignant tissues compared with adenomas or non-tumorous tissues of the colon [14, 15].

The redox-regulated multifunctional protein DJ-1 is involved in diverse cell processes such as chemotaxis, cell migration, cell adhesion, angiogenesis, apoptosis, cell–extracellular matrix interactions and immune regulation [16]. It protects cells from oxidative stress, detoxifies reactive oxygen species and is converted into a variant with a more acidic isoelectric point [16]. Overexpression of DJ-1 increases the chemoresistance of neoplastic cells by inhibiting apoptotic pathways [16, 17]. It has been associated with early-onset parkinsonism [18], hence its other name PARK7, and it is overexpressed in several malignant tumors including breast, lung, glottic and esophageal carcinomas [19–22]. Increased serum DJ-1 levels have been reported in patients with metastatic uveal melanoma, pancreatic cancer and endometrioid-type endometrial cancers (EECs) [23–25].

We have previously reported that high serum and tumor tissue levels of 8-OHdG in epithelial ovarian carcinoma (EOC) patients are associated with traditional factors of poor prognosis and also with serous histology [26]. In this previous study, 75 % of patients had serous histology and we were therefore unable to assess the prognostic value in different histological subtypes. We included 35 patients from the previous study to the current study (32 serous, 2 endometrioid and 1 clear cell histology) aiming to evaluate the roles of serum 8-OHdG and DJ-1 as prognostic factors in different histological types of epithelial ovarian cancer.

## Methods

The study involved 112 epithelial ovarian cancer patients diagnosed and treated at Oulu University Hospital in 1996–2009. In most cases, the patients were operated upon at Oulu University Hospital. In some cases the primary operation was in the central hospital, as the tumor was assumed to be benign. In those cases, the staging operation was carried out at Oulu University Hospital. Operations were categorized as optimal if no macroscopic tumor remained and suboptimal if there was any residual tumor. After the operation, the patients underwent taxane- and platinum-based chemotherapy for a total of six treatments at three-week intervals. If the patients showed a complete response after chemotherapy, they were transferred to a routine follow-up program.

The patient data was retrospectively collected from the archives. Age, histology, grade, stage, optimality of the operation, resistance to platinum-based chemotherapy, disease-free survival (DFS) and overall survival (OS) were recorded (Table 1). Recurrence was diagnosed according to radiological RECIST 1.1 criteria [27]. Platinum resistance was defined as progression during platinum treatment or recurrence <  6 months after the last platinum dose. Overall survival was calculated as the time from diagnosis to the time of death.

**Table 1:** Patient characteristics

Histology	
Serous	38 (33.9 %)
Mucinous	20 (17.9 %)
Endometrioid	34 (30.4 %)
Clear-cell	20 (17.9 %)
Age	22–85 (mean 59)
Stage	
I–II	50 (44.6 %)
III–IV	60 (53.6 %)
Missing	2 (1.8 %)
Grade	
1	22 (19.6 %)
2	17 (15.2 %)
3	71 (63.4 %)
Missing	2 (1.8 %)
Surgical Outcome	
Optimal	55 (49.1 %)
Suboptimal	21 (18.8 %)
Inoperable	35 (31.3 %)
Missing	1 (0.9 %)
Platinum resistance	
Yes	43 (38.4 %)
No	48 (42.9 %)
Other Reason	2 (1.8 %)
Missing	19 (17.0 %)
Recurrence	
Yes	58 (51.8 %)
No	51 (45.5 %)
Missing	3 (2.7 %)

Serum samples were collected from every patient the day before the operation or at the time of diagnosis and the samples were stored at −70 °C until use. Commercial ELISA kits for assay of 8-OHdG (JaICA, Japan) and DJ-1 (Nordic BioSite, Sweden) were used. The assays were carried out by following the manufacturers’ instructions, except that sample filtering was omitted.

The data was analyzed by using IBM SPSS Statistics for Windows 21.0.0.0 software. The significance of associations was assessed by using the Mann–Whitney *U* test and the Kruskal–Wallis test. In survival analysis, serum concentrations were re-formatted as two-class variables using the median level as a cut-off point. Survival was analyzed by using Kaplan–Meier curves and the log-rank test. Cox multivariate regression analysis was used for multivariate analysis. Probability values < 0.05 were considered significant.

This study was approved by the National Supervisory Authority for Welfare and Health (1339/05.01.00.06/2009) and The Regional Ethics Committee of the Northern Ostrobothnia Hospital District (53/2008).

## Results

### Correlation with traditional EOC risk factors

**Fig. 1:**
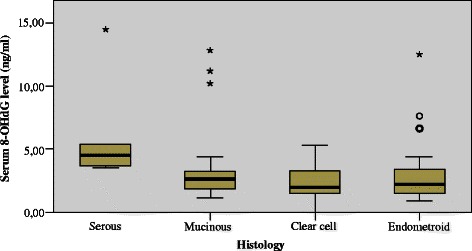
Association between EOC histology and serum 8-OHdG level (p = 0.033)

Serum levels of 8-OHdG were determined in 105 samples and the mean concentration was 3.22 ng/ml (95 % CI 2.73–3.71 ng/ml). The median 8-OHdG concentration was 2.53 ng/ml and this cut-off level was applied in Kaplan–Meier analysis. The mean concentration of DJ-1 (n = 48) was 15.99 ng/ml (95 % CI 12.2–19.8 ng/ml), the median being 10.5 ng/ml. A high level of 8-OHdG was associated with serous histology vs. other types (p = 0.033), platinum resistance (p = 0.002), stage III–IV versus I–II (p = 0.009) and a suboptimal surgical outcome (p = 0.012) (Fig. 1, Table 2). Among the patients with endometrioid histology, a high serum 8-OHdG level was associated with platinum resistance (p = 0.007) and a suboptimal surgical outcome (p = 0.025).

**Table 2:** Associations between prognostic factors and serum concentrations of 8-OHdG in the whole study population, in the endometrioid subgroup, and levels of DJ-1

	Serous histology	Platinum resistance	Stage III–IV	Inoperability	Grade
High 8-OHdG	0.033	0.002	0.009	0.012	NS
High 8-OHdG	NS	0.007	NS	0.025	NS
Endometrioid histology					
High DJ-1	0.089	NS	NS	NS	0.029

Values are p-values, NS = non-significant

High DJ-1 levels were significantly associated with a high histological grade (p = 0.029) and non-significantly associated with serous histology versus other histology (p = 0.089). No associations between levels of 8-OHdG and the stage of the disease, or 8-OHdG and DJ-1 levels were found.

### Survival analysis

**Fig. 2:**
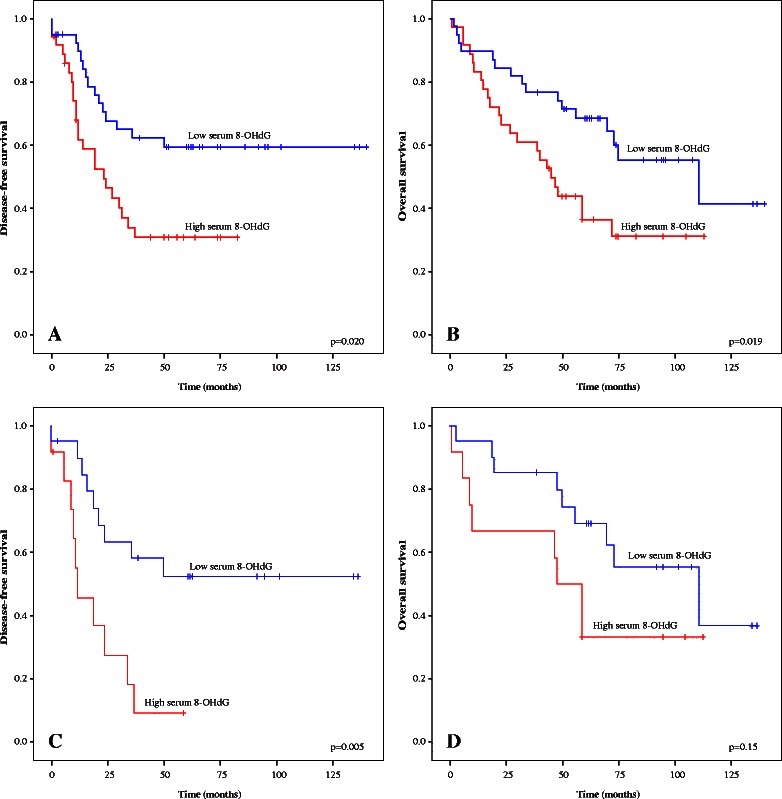
Kaplan–Meier curves demonstrating relationships between serum 8-OHdG concentrations and disease-free survival in the whole study population (**a**), overall survival in the whole study population (**b**), disease-free survival in cases of carcinoma with endometrioid histology (**c**) and overall survival in the endometrioid histology group (**d**)

Higher 8-OHdG concentrations were associated with poor DFS (p = 0.020) as well as with poor OS (p = 0.019) in the whole study group (Fig. 2). In cases of endometrioid histology, serum 8-OHdG levels were highly significantly associated with DFS (p = 0.005).

In Cox regression analysis a high level of serum 8-OHdG in the patients with endometrioid histology was a more significant predictor of poor DFS (OR 1.387; 95 % CI 1.095–1.758; p = 0.007) than a suboptimal surgical result (OR 2.898; 95 % CI 0.925–9.076; p = 0.068), high stage (OR 1.221; 95 % CI 0.215–6.915, p = 0.822) or high histological grade (OR 1.566; 95 % CI 0.259–9.457, p = 0.625).

## Discussion

We report here that the preoperative serum concentration of 8-OHdG is a highly significant prognostic marker in cases of EOC, especially in endometrioid ovarian carcinomas. On the other hand, serum DJ-1 levels seem not to have prognostic significance in cases of EOC.

As one of the most studied oxidative stress markers, 8-OHdG has been investigated by way of immunohistochemistry in connection with various carcinomas, but via serum 8-OHdG levels in only a few cancer types. The main repair enzyme connected with 8-OHdG is (human) 8-oxoguanine DNA glycosylase 1 (hOGG1), which cleaves damaged guanosine from DNA and thereafter it is secreted to extracellular fluids [28]. The existing data suggest that extracellular 8-OHdG levels are not affected by diet, cell death or artifact formation [29]. High serum 8-OHdG levels have been recognized as a predictor of short-term survival in non-small-cell lung cancer patients [11], serous ovarian cancer patients [26, 30] and in children with acute leukemia [31]. In one of our previous studies [26] we reported that high serum 8-OHdG levels as well as high 8-OHdG immunohistochemical expression were associated with poor clinical outcome in cases of serous ovarian cancer. However, that particular study was focused mainly on serous carcinomas, serum 8-OHdG associated to poor survival only in low grade disease, we were not able to assess progression-free survival and prognostic significance was not observed in multivariate analysis. On the basis of the results, we suggested that there could be variation in oxidative stress levels between different ovarian cancer histological subtypes, and 8-OHdG levels could possibly reflect chemoresistance – hence the current study was undertaken.

We have previously reported higher 8-OHdG tissue expression in borderline ovarian tumors than in benign ones, suggesting a role of 8-OHdG in early ovarian epithelial carcinogenesis [32]. Epithelial ovarian cancers can be assigned to different groups not only by their different histological appearances but also by their obviously different carcinogenetic patterns. In this study serous carcinomas were associated with the highest serum 8-OHdG levels, but the endometrioid histotype was the one where the most significant association between serum 8-OHdG levels and the outcome of the disease was observed. Some endometrioid ovarian cancers are known to arise from atypical endometriosis. We have previously shown biochemical connections between endometriosis and endometriosis-associated endometrioid cancers. In those studies immunohistochemical 8-OHdG expression in endometrioid cancer tissue was significantly lower than in the related endometriosis tissue [33]. This finding suggests that at a certain phase of cancer development, DNA guanine adducts are degraded at the tissue level and 8-OHdG is released to the serum. Thus, 8-OHdG might be a surrogate marker of induced cancer metabolism.

One of the main findings in this study was the association between preoperative serum 8-OHdG levels, not only with all studied traditional prognostic factors, but also with the earlier development of chemoresistance. One of the most important mechanisms of action of platinum regimens used in cases of ovarian carcinoma is based on ROS generation. Therefore, we cannot rule out the possibility that the association with chemoresistance could be explained by the induction of antioxidant enzymes in the most oxidatively stressed tumors. Our observation of a lack of association between serum concentrations of 8-OHdG and DJ-1 is not necessarily contradictory to this hypothesis, since there are also DJ-1-independent redox-regulating pathways such as Nrf2, which recently was connected to platinum resistance in cases of bladder cancer [34]. On the other hand, elevated levels of ROS may simply generate mutations that are able to favor the survival of some cancer cell subpopulations.

High disease burden, poor differentiation and suboptimal surgical outcome are well characterized prognostic factors in all subtypes of ovarian cancer. However, an elevated serum 8-OHdG level was an even more powerful predictor of disease relapse, but only in connection with endometrioid histology. Although it did not significantly reflect OS in multivariate analysis, DFS is a valid endpoint from a clinical point of view. The median survival time of the patients with an endometrioid histotype and high 8-OHdG serum concentrations was 48 months, compared with 111 months in the endometrioid ovarian cancer patients with low 8-OHdG serum levels. In clear-cell and mucinous cancer types the serum 8-OHdG level was not of prognostic value in this patient cohort.

DJ-1 is an oxidative response protein with oncogenic properties. It induces PI3K-AKT and ERK1/3 signaling, influences c-myc expression and provides protection from ROS-induced apoptosis *in vitro* [35–37]. Increased serum DJ-1 levels have been detected in endometrioid-type endometrial cancer (EEC) patients compared with healthy controls [25]. In the same study, higher DJ-1 serum levels and immunohistochemical expression in the serous type of endometrial cancer (ESC) were observed when set against the levels in cases of EEC. In ovarian cancer, DJ-1 levels, measured by PCR from effusions, were more expressed at an advanced stage [38]. In gastric carcinoma DJ-1 plays an important role in the development of peritoneal carcinomatosis *in vitro* [39] and in breast cancer low DJ-1 immunohistochemical and FISH (fluorescence *in situ* hybridization) expression seems to predict pathological complete remission after neoadjuvant chemotherapy [40]. High DJ-1 immunohistochemical expression in pancreatic cancer is associated with chemoresistance [24]. In prostate carcinoma tissue DJ-1 is overexpressed compared with that in benign hyperplasia [41], and in non-small-cell lung cancer overexpression has been described compared with that in normal lung tissue [42]. In the current material elevated DJ-1 concentrations were observed in patients with high-grade EOC, but no associations with other traditional prognostic factors or survival were found.

The present results confirm the hypothesis that different epithelial ovarian cancer histotypes represent different carcinogenic backgrounds and clinical behavior and they should be categorized in more detail according to their biomolecular characteristics. Serum concentrations of 8-OHdG also predict the development of platinum resistance and could thus assist in the choice of chemotherapy. This is in line with the results of our previous clinical pilot study of biomarkers of the response to bevacizumab in serous ovarian cancer. In that study, immunohistochemical expression of 8-OHdG appeared to predict a longer-term response to bevacizumab treatment [43].

## Conclusions

An elevated serum 8-OHdG level is a highly significant predictor of poor prognosis, especially in cases of the endometrioid subtype of epithelial ovarian cancer. In addition, high 8-OHdG levels predict earlier development of platinum resistance, which may have a significant clinical impact if confirmed in future studies. These results could be beneficial as regards chemotherapy and maintenance treatment in cases of ovarian cancer.

## References

[1] Murdoch WJ, Martinchick JF (2004). Oxidative damage to DNA of ovarian surface epithelial cells affected by ovulation: carcinogenic implication and chemoprevention. Exp Biol Med.

[2] Murdoch WJ (2005). Carcinogenic potential of ovulatory genotoxity. Biol Reprod.

[3] Murdoch WJ, Van Kirk EA, Alexander BM (2005). Damages in ovarian surface epithelial cells of ovulatory hens. Exp Biol Med.

[4] Ness RB, Cottreau C (1999). Possible role of ovarian epithelial inflammation in ovarian cancer. J Natl Cancer Inst.

[5] Yang-Hartwich Y, Gurrea-Soteras M, Sumi N, Joo WD, Holmberg JC, Craveiro V, et al. Ovulation and extra-ovarian origin of ovarian cancer. Sci Rep. 2014; doi:10.1038/srep06116. 10.1038/srep06116 PMC4137344 25135607

[6] Valko M, Izakovic M, Mazur M, Rhodes CJ, Telser J (2004). Role of oxygen radicals in DNA damage and cancer incidence. Mol Cell Biochem.

[7] Karihtala P, Soini Y, Vaskivuo L, Bloigu R, Puistola U (2009). DNA adduct 8-hydroxydeoxyguanosine, a novel putative marker of prognostic significance in ovarian carcinoma. Int J Gynecol Cancer.

[8] Sova H, Jukkola-Vuorinen A, Puistola U, Kauppila S, Karihtala P (2010). 8-Hydroxydeoxyguanosine: a new potential independent prognostic factor in breast cancer. Br J Cancer.

[9] Sheridan J, Wang LM, Tosetto M, Sheahan K, Hyland J, Fennelly D, O`Donoghue D, Mulcahy H, O`Sullivan J (2009). Nuclear oxidative damage correlates with poor survival in colorectal cancer. Br J Cancer.

[10] Murtas D, Piras F, Minerba L, Ugalde J, Floris C, Maxia C, Demurtas P, Perra MT, Sirigu P (2010). Nuclear 8-hydroxy-2`-deoxyguanosine, a survival biomarker in patients with cutaneous melanoma. Oncol Rep.

[11] Shen J, Deiniger P, Hunt JD, Zhao H (2007). 8-Hydroxy-2`-deoxyguanosine (8-OH-dG) as a potential survival biomarker in patients with non-small-cell lung cancer. Cancer.

[12] Peroja P, Pasanen AK, Haapasaari KM, Jantunen E, Soini Y, Turpeenniemi-Hujanen T, et al. Oxidative stress and redox state-regulating enzymes have prognostic relevance in diffuse large B- cell lymphoma. Exp Hematol Oncol. 2012; doi:10.1186/2161-3619-1-2. 10.1186/2162-3619-1-2 PMC3506993 23210982

[13] He H, Zhao Y, Wang N, Zhang L, Wang C (2014). 8-Hydroxy-2`-deoxyguanosine expression predicts outcome of esophageal cancer. Ann Diagn Pathol.

[14] Kondo S, Toyokuni S, Iwasa Y (1999). Persistent oxidative stress in human colorectal carcinoma, but not in adenoma. Free Radic Biol Med.

[15] Murawaki Y, Tsuchiya H, Kanbe T, Harada K, Yashima K, Nozaka K, Tanida O, Kohno M, Mukiyama T, Nishimuki R, Kojo H, Matsura T, Takahashi K, Osaki M, Ito H, Yodoi J, Murawaki Y, Shiota G (2008). Aberrant expression of selenoproteins in the progression of colorectal cancer. Cancer Lett.

[16] Junn E, Taniguchi H, Jeong BS, Zhao X, Ichijo H, Mouradian MM (2005). Interaction of DJ-1 with Daxx inhibits apoptosis signal-regulating kinase 1 activity and cell death. Proc Natl Acad Sci U S A.

[17] Kim RH, Peters M, Jang Y, Shi W, Pintilie M, Fletcher GC, DeLuca C, Liepa J, Zhou L, Snow B, Binari RC, Manoukian AS, Bray MR, Liu FF, Tsao MS, Mak TW (2005). DJ-1, a novel regulator of the tumor suppressor PTEN. Cancer Cell.

[18] Bonifati V, Rizzu P, Squitieri F, Krieger E, Vanacore N, van Swieten JC, Brice A, van Duijn CM, Oostra B, Meco G, Heutink P (2003). DJ-1 (PARK7), a novel gene for autosomal recessive, early onset parkinsonism. Neurol Sci.

[19] Merikallio H, Pääkkö P, Kinnula VL, Harju T, Soini Y (2012). Nuclear factor erythroid-derived 2-like 2 (Nrf2) and DJ1 are prognostic factors in lung cancer. Hum Pathol.

[20] Le Naour F, Misek DE, Krause MC, Deneux L, Giordano TJ, Scholl S, Hanash SM (2001). Proteomics-based identification of RS/DJ-1 as a novel circulating tumor antigen in breast cancer. Clin Cancer Res.

[21] Zhu XL, Wang ZF, Lei WB, Zhuang HW, Jiang HY, Wen WP (2010). DJ-1: a novel independent prognostic marker for survival in glottic squamous cell carcinoma. Cancer Sci.

[22] Yuen HF, Chan YP, Law S, Srivastava G, El-Tanani M, Mak TW, Chan KW (2008). DJ-1 could predict worse prognosis in esophageal squamous cell carcinoma. Cancer Epidemiol Biomarkers Prev.

[23] Chen LL, Tian JJ, Su L, Jing Y, Zhang SC, Zhang HX, Wang XQ, Zhu CB (2015). DJ-1: a promising marker in metastatic uveal melanoma. J Cancer Res Clin Oncol.

[24] Tsiaousidou A, Lambropoulou M, Chatzitheoklitos E, Tripsiais G, Tsompanidou C, Simopoulos C, Tsaroucha AK (2013). B7H4, HSP27 and DJ-1 molecular markers as prognostic factors in pancreatic cancer. Pancreatology.

[25] Morelli M, Scumaci D, Di Cello A, Venturella R, Donato G, Faniello MC, Quaresima B, Cuda G, Zullo F, Costanzo F (2014). DJ-1 in endometrial cancer: a possible biomarker to improve differential diagnosis between subtypes. Int J Gynecol Cancer.

[26] Pylväs M, Puistola U, Laatio L, Kauppila S, Karihtala P (2011). Elevated serum 8-OHdG is associated with poor prognosis in epithelial ovarian cancer. Anticancer Res.

[27] Eisenhauer EA, Therasse P, Bogaerts J, Schwartz LH, Sargent D, Ford R, Dancey J, Arbuck S, Gwyther S, Mooney M, Rubinstein L, Shankar L, Dodd L, Kaplan R, Lacombe D, Verweij J (2009). New response evaluation criteria in solid tumours: Revised RECIST guideline (version 1.1). Eur J Cancer.

[28] Hirano T (2008). Repair system of 7, 8-dihydro-8-oxoguanine as a defense line against carcinogenesis. J Radiat Res.

[29] Cooke MS, Evans MD, Dove R, Rozalski R, Gackowski D, Siomek A, Lunec J, Olinski R (2005). DNA repair is responsible for the presence of oxidatively damaged DNA lesions in urine. Mutat Res.

[30] Xu X, Wang Y, Guo W, Zhou Y, Lv C, Chen X, et al. The significance of the alteration of 8-OHdG in serous ovarian carcinoma. J Ovarian Res. 2013;doi:10.1186/1757-2215-6-74. 10.1186/1757-2215-6-74 PMC3875897 24165045

[31] Valavanidis A, Viachogianni T, Fiotakis C (2009). 8-Hydroxy-2-deoxyguanisine (8-OHdG): A critical biomarker of oxidative stress and carcinogenesis. J Environ Sci Health C Environ Carcinog Exotoxicol Rev.

[32] Pylväs M, Puistola U, Kauppila S, Karihtala P (2010). Oxidative stress-induced antioxidant enzyme expression is an early phenomenon in ovarian carcinogenesis. Eur J Cancer.

[33] Sova H, Kangas J, Puistola U, Santala M, Liakka A, Karihtala P (2012). Down-regulation of 8-hydroxyguanosine and peroxiredoxin II in the pathogenesis of endometriosis-associated ovarian cancer. Anticancer Res.

[34] Hayden A, Douglas J, Sommerland M, Andrews L, Gould K, Hussain S, Thomas GJ, Packham G, Crabb SJ (2014). The Nrf2 transcription factor contributes to resistance to cisplatin in bladder cancer. Urol Oncol.

[35] Gu L, Cui T, Fan C, Zhao H, Zhao C, Lu L, Yang H (2009). Involvement of ERK1/2 signaling pathway in DJ-1-induced neuroprotection against oxidative stress. Biochem Biophys Res Commun.

[36] Ismail IA, Kang HS, Lee HJ, Kwon BM, Hong SH (2012). 2'-Benzoyloxycinnamaldehyde-mediated DJ-1 upregulation protects MCF-7 cells from mitochondrial damage. Biol Pharm Bull.

[37] Inberg A, Linial M (2010). Protection of pancreatic beta-cells from various stress conditions is mediated by DJ-1. J Biol Chem.

[38] Davidson B, Hadar R, Schlossberg A, Sternlicht T, Slipicevic A, Skrede M, Risberg B, Flørenes VA, Kopolovic J, Reich R (2008). Expression and clinical role of DJ-1, a negative regulator of PTEN, in ovarian carcinoma. Hum Pathol.

[39] Zhu ZM, Li ZR, Huang Y, Yu HH, Huang XS, Shao JH, Chen HP (2014). DJ-1 is involved in the peritoneal metastasis of gastric cancer through activation of the Akt signaling pathway. Oncol Rep.

[40] Kawate T, Iwaya K, Kikuchi R, Kaise H, Oda M, Sato E, Hiroi S, Matsubara O, Kohno N (2013). DJ-1 protein expression as a predictor of pathological complete remission after neoadjuvant chemotherapy in breast cancer patients. Breast Cancer Res Treat.

[41] Osman WM, Abd El Atti RM, Abou Gabal HH (2013). DJ-1 and androgen receptor immunohistochemical expression in prostatic carcinoma: a possible role in carcinogenesis. J Egypt Natl Canc Inst.

[42] Bai J, Guo C, Sun W, Li M, Meng X, Yu Y, Jin Y, Tong D, Geng J, Huang Q, Qi J, Fu S (2012). DJ-1 may contribute to metastasis of non-small cell lung cancer. Mol Biol Rep.

[43] Karihtala P, Mäenpää J, Turpeenniemi-Hujanen T, Puistola U (2010). Front-line bevacizumab in serous epithelial ovarian cancer: biomarker analysis of the FINAVAST trial. Anticancer Res.

